# Guided Implant Surgery in Oral Cancer Patients: Initial Clinical Experience from an Academic Point-of-Care Manufacturing Unit

**DOI:** 10.3390/medicina62010151

**Published:** 2026-01-12

**Authors:** Manuel Tousidonis, Jose-Ignacio Salmeron, Santiago Ochandiano, Ruben Perez-Mañanes, Estela Gomez-Larren, Elena Aguilera-Jimenez, Carla de Gregorio-Bermejo, Diego Fernández-Acosta, Borja Gonzalez-Moure, Saad Khayat, Carlos Navarro-Cuellar

**Affiliations:** 1 Department of Oral and Maxillofacial Surgery, Gregorio Marañon University Hospital, 28007 Madrid, Spain; joseignacio.salmeron@salud.madrid.org (J.-I.S.); santiago.ochandiano@salud.madrid.org (S.O.); dfacosta@salud.madrid.org (D.F.-A.); bgmoure@salud.madrid.org (B.G.-M.); saad.khayat@salud.madrid.org (S.K.); 2 Gregorio Marañón Research Institute, 28007 Madrid, Spain; ruben.perez@salud.madrid.org (R.P.-M.); egomezlarren.externo@salud.madrid.org (E.G.-L.); elena.aguilera.externo@salud.madrid.org (E.A.-J.); carla.gregorio.externo@salud.madrid.org (C.d.G.-B.); 3 Advanced Planning and 3D Manufacturing Unit (UPAM3D), Hospital General Universitario Gregorio Marañón, Dr. Esquerdo 46, 28007 Madrid, Spain; 4 Department of Surgery, Universidad Complutense de Madrid, 28040 Madrid, Spain

**Keywords:** point-of-care manufacturing, static guided implant surgery, dental implant, oral cancer, implant rehabilitation, 3D printing, maxillofacial surgery, additive manufacturing, head and neck cancer, reconstruction

## Abstract

*Background and Objectives*: Implant-supported rehabilitation after oral cancer surgery remains technically and biologically demanding due to altered anatomy, scar tissue, and prior radiotherapy. Digital workflows and hospital-based point-of-care (POC) manufacturing now enable personalized, prosthetically driven implant placement with static surgical guides fabricated within the clinical environment. This study reports the initial clinical experience of an academic POC manufacturing unit (UPAM3D) implementing static guided implant surgery in oral cancer patients and compares this approach with conventional outsourcing and dynamic navigation methods. *Materials and Methods*: A retrospective review of 30 consecutive cases (2021–2024) treated with POC-manufactured static guides was conducted using data from the UPAM3D registry. Each record included design, fabrication, and sterilization parameters compliant with ISO 13485 standards. Demographic, surgical, and prosthetic variables were analyzed, including anatomical site (maxilla or mandible), guide type, material, radiotherapy history, number of Ticare Implants^®^, and loading strategy. *Results*: All surgical guides were designed and 3D printed in-house using biocompatible resins (BioMed Clear, Dental SG, or LT Clear). The annual number of POC procedures increased progressively (2 → 6 → 6 → 16). Most cases involved oncologic reconstructions of the maxilla or mandible, including irradiated fields. When recorded, primary stability values (mean ISQ ≈ 79) allowed immediate or early loading (ISQ ≥ 70). No major intraoperative or postoperative complications occurred, and all guides met sterilization and traceability standards. *Conclusions*: Point-of-care manufacturing enables efficient, accurate, and patient-specific guided implant rehabilitation after oral cancer surgery, optimizing functional and esthetic outcomes while reducing procedural time and dependence on external providers. Integrating this process into clinical workflows supports personalized treatment planning and broadens access to advanced implant reconstruction within multidisciplinary oncology care.

## 1. Introduction

Rehabilitation of patients after oral cancer treatment remains one of the most demanding challenges in maxillofacial surgery [1]. Beyond tumor removal and flap viability, long-term goals include restoring mastication, speech, and facial esthetics—factors that directly affect quality of life and psychosocial reintegration [2]. Conventional implant placement is often complicated by anatomical alterations, bone discontinuities, fibrosis, and limited soft-tissue mobility, especially in irradiated patients [3]. As a result, freehand techniques may lead to suboptimal implant positioning, longer operative times, and difficulties in achieving prosthetically guided rehabilitation [4]. With improving survival rates, the focus has shifted toward functional and esthetic restoration through more precise, reproducible, and efficient implant-based reconstruction methods [5].

Over the past two decades, digital planning has transformed maxillofacial surgery. The integration of virtual surgical planning (VSP), computer-aided design and manufacturing (CAD/CAM), and additive manufacturing (AM) enables the simulation, design, and execution of complex procedures with high accuracy [6]. This digital transformation introduced two main navigation approaches for implant placement: static guided surgery, which transfers a virtual plan to the patient via a prefabricated guide, and dynamic navigation, which provides real-time optical tracking feedback [7,8]. Static guidance ensures consistent precision and eliminates intraoperative calibration, whereas dynamic systems allow on-the-fly adjustments. In oncologic cases—where anatomy is distorted and radiotherapy or flap reconstructions complicate navigation—static guides are often favored for reducing operative time and ensuring prosthetically driven implant positioning [9,10,11].

Digital workflows have naturally evolved toward the concept of point-of-care (POC) manufacturing, in which medical devices are designed and fabricated directly within the hospital under a certified quality system (ISO 13485) and the European regulatory framework (EU MDR 2017/745) [12,13,14,15,16]. Unlike outsourcing to industrial suppliers, POC manufacturing allows immediate surgeon–engineer collaboration, rapid turnaround, and full traceability of materials and processes [12]. In academic hospitals, this integration facilitates quick iteration between design and surgery, producing patient-specific solutions tailored to each anatomy and treatment schedule. Since 2018, the UPAM3D unit at Hospital General Universitario Gregorio Marañón has functioned as a certified POC facility, manufacturing patient-specific devices for cranio-maxillofacial surgery—including anatomical models, resection guides, implants, and splints [14]. Its integration within the clinical workflow ensures a seamless transition from planning to manufacturing, maintaining validated sterilization and quality assurance without external dependence.

In oral oncology, POC manufacturing offers distinct advantages. Patients undergoing segmental resections or complex reconstructions often need customized rehabilitation strategies because of limited bone stock, compromised vascularization, or previous irradiation. Designing and producing surgical guides within the hospital enables rapid adaptation to anatomic and scheduling constraints, even for urgent or multi-stage procedures [17]. The availability of biocompatible stereolithographic resins (BioMed Clear, Dental SG, and LT Clear), validated for steam sterilization at 134 °C, makes it possible to fabricate precise and safe implant guides for intraoperative use [12,15,18]. Combined with intraoperative CT or structured-light scanning, this ecosystem provides a reproducible and auditable framework for precision surgery [13,19,20,21,22].

Therefore, this study aims to (1) describe the implementation and evolution of the POC workflow between 2021 and 2024; (2) analyze design, manufacturing, and clinical parameters, including implant stability and early outcomes; and (3) discuss the advantages and limitations of POC manufacturing compared with conventional and computer-assisted techniques.

## 2. Materials and Methods

### 2.1. Study Design and Setting

This retrospective observational study was conducted at the Department of Oral and Maxillofacial Surgery, Hospital General Universitario Gregorio Marañón (Madrid, Spain). Data were obtained from the UPAM3D registry—which prospectively records all in-house manufactured medical devices—and from the hospital’s electronic health records. The study followed STROBE recommendations for observational research.

Inclusion criteria were (1) patients with oral cancer or oncologic sequelae who received Ticare Implants^®^ (MOZO-GRAU, S.A., Valladolid, Spain); (2) use of a static surgical guide fabricated entirely in-house by the UPAM3D unit; and (3) availability of postoperative CT or CBCT imaging and at least six months of clinical follow-up.

Exclusion criteria were incomplete design documentation, non-guided implant placement, or use of externally manufactured guides.

This analysis included all consecutive patients treated with POC-manufactured static guides for oral oncologic rehabilitation between January 2021 and December 2024, corresponding to the full period since implementation of the UPAM3D guided implant workflow in our institution. No additional case selection was performed beyond the predefined inclusion and exclusion criteria.

### 2.2. Point-of-Care (POC) Workflow

The standardized UPAM3D manufacturing process followed a validated ISO 13485–compliant protocol comprising the following sequential phases:

1. Imaging: acquisition of preoperative CT or CBCT scans (voxel size ≤ 0.4 mm) in DICOM format.
2. Segmentation and planning: creation of three-dimensional anatomical models using open-source or commercial software (3D Slicer version 5.2.2, Materialise 3-matic version 20.0Blender version 2.75 (Blender Foundation)). Implant positioning was prosthetically driven and jointly verified by the surgeon and engineer.
3. Guide design: creation of bone-, tooth-, or mucosa-supported templates incorporating metallic sleeves or printed guidance channels.
4. In-house manufacturing: stereolithographic printing on Form 3B/3BL systems (Formlabs Inc., Somerville, MA 02143, USA) using Class I biocompatible resins (BioMed Clear, Dental SG, or LT Clear).
5. Post-processing: double washing in isopropyl alcohol (≥96%), UV curing (405 nm), mechanical trimming, and visual surface inspection.
6. Packaging and sterilization: individual sealing and steam sterilization at 134 °C for 5 min, validated by routine biological indicators and ISO 17665 documentation.
7. Intraoperative placement: guided implant insertion according to the prosthetically driven plan.
8. Primary stability measurement: evaluation of implant stability with Osstell^®^ resonance frequency analysis (Implant Stability Quotient, ISQ) to determine immediate or early loading potential.
9. Postoperative verification: intraoperative or postoperative CBCT or structured-light scanning to assess implant positioning accuracy.

Each stage was recorded in a digital traceability sheet that documented patient code, design approval, operator, material batch number, printer identification, and sterilization lot. All devices were stored in the institutional POC quality management database for auditability.

### 2.3. Data Collection

The following variables were collected: demographic data (age, sex), diagnosis, anatomical site (maxilla or mandible), reconstruction type (native bone, fibula, iliac, or scapular flap), radiotherapy history, number of Ticare Implants^®^, guide type and material, design-to-surgery interval, ISQ values, loading protocol (immediate, early, or delayed), and any complications. Radiological verification was used to assess coronal, apical, and angular deviations from the preoperative plan whenever available. Clinical outcomes included implant survival and time to definitive prosthesis delivery.

All data were anonymized prior to analysis and entered into a password-protected spreadsheet stored on the institutional network.

### 2.4. Data Analysis

Descriptive analyses were performed using SPSS (version 29.0, IBM Corp.) and Microsoft Excel. Continuous variables are presented as mean ± standard deviation (SD) or as median and range, as appropriate. Categorical variables were summarized as absolute and relative frequencies. Temporal trends were assessed graphically. Because of the exploratory nature of this study, no hypothesis testing or inferential statistics were performed.

### 2.5. Ethical Considerations

The study was conducted in accordance with the Declaration of Helsinki (2013 revision) and the institutional policy for retrospective analyses of anonymized data. Ethical approval was granted by the Clinical Research Ethics Committee of Hospital General Universitario Gregorio Marañón (protocol code: impCMF01—3 November 2023). All devices were designed and manufactured under UPAM3D’s CE-marked, ISO 13485–certified POC framework. Informed consent for the use of anonymized clinical and imaging data was obtained at the time of treatment.

### 2.6. Data and Material Availability

The datasets and manufacturing parameters supporting this study are available from the corresponding author upon reasonable request and institutional approval. No proprietary code or restricted software was used. All materials (resins, printers, sterilization methods) are commercially available and listed herein.

### 2.7. Use of Generative Artificial Intelligence

During the preparation of this manuscript, ChatGPT (OpenAI, GPT-5, 2025 edition) was used solely for language editing and formatting under the supervision of the authors. No AI tool was employed for data generation, analysis, or interpretation. The authors reviewed and verified all content for accuracy and scientific integrity.

## 3. Results

### 3.1. General Overview

**Figure 1:**
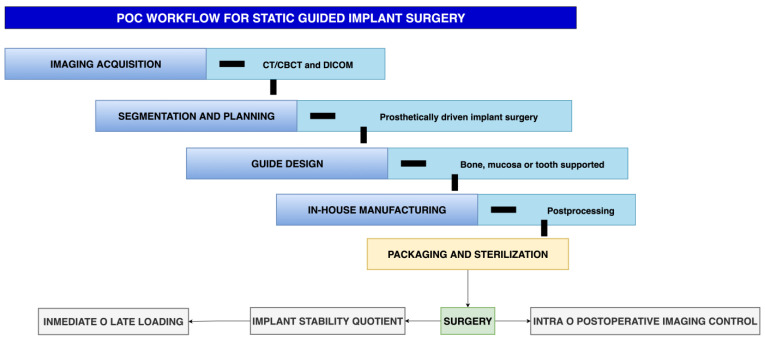
Point-of-care (POC) workflow for static guided implant surgery. The process begins with imaging acquisition (CT/CBCT and DICOM data), followed by segmentation and prosthetically driven planning, guide design (bone-, mucosa-, or tooth-supported), and in-house manufacturing with postprocessing, packaging, and sterilization. Surgery is performed with intra- or postoperative imaging control, implant stability assessment (ISQ), and immediate or delayed loading depending on primary stability.

A total of 30 static surgical guides were designed, manufactured, and applied for implant-supported rehabilitation using the point-of-care (POC) workflow at the UPAM3D unit. These guides enabled the placement of 148 Ticare Implants^®^ between 2021 and 2024. The steady increase in annual cases—from two in 2021 to sixteen in 2024—reflects the successful integration of the POC workflow into routine clinical practice (Figure 1).

Patients had a mean age of 58 years (range 42–77), with 19 males and 11 females. All cases involved oral oncologic surgery, performed either in reconstructed jaws (70%) or in native bone after segmental resection. Over the study period, static guided surgery progressively replaced freehand or outsourced workflows as the institutional standard for oncologic implant rehabilitation.

### 3.2. Anatomic and Oncologic Distribution

Implant placement sites included the mandible (17 guides, 57%) and maxilla (13 guides, 43%). Twenty-two guides (73%) were used in microvascular or preprosthetic reconstructions—primarily fibula (n = 20) and iliac crest (n = 2). Nine procedures (30%) involved irradiated bone segments with cumulative doses ≥50 Gy.

The most frequent underlying diagnosis was squamous cell carcinoma (n = 18 guides, 60%), followed by ameloblastoma and other benign aggressive tumors (n = 9, 30%), and miscellaneous conditions such as condrosarcoma, osteoradionecrosis or reconstructive sequelae (n = 3, 10%).

Baseline characteristics and workflow parameters are summarized in Table 1.

### 3.3. Design and Manufacturing Parameters

All 30 guides were digitally designed through a collaborative surgeon–engineer workflow using virtual surgical planning (VSP) software (3D Slicer, Blender, or Materialise 3-matic). Each design underwent at least two validation checkpoints to confirm prosthetically driven implant alignment and optimal guide support.

Guides were fabricated in-house using stereolithography (Form 3B/3BL; Formlabs Inc., USA) with certified biocompatible resins—BioMed Clear (57%), Dental SG (33%), and LT Clear (10%). Support types included bone-based (18 guides), tooth-based (7), and mucosa-based (5).

Each device was post-processed by sequential isopropyl alcohol baths, UV curing, mechanical trimming, and visual inspection. Steam sterilization (134 °C, 5 min) was validated under ISO 13485. Every guide was assigned a unique batch number ensuring full traceability, including operator ID, resin lot, and printer serial number.

**Figure 2:**
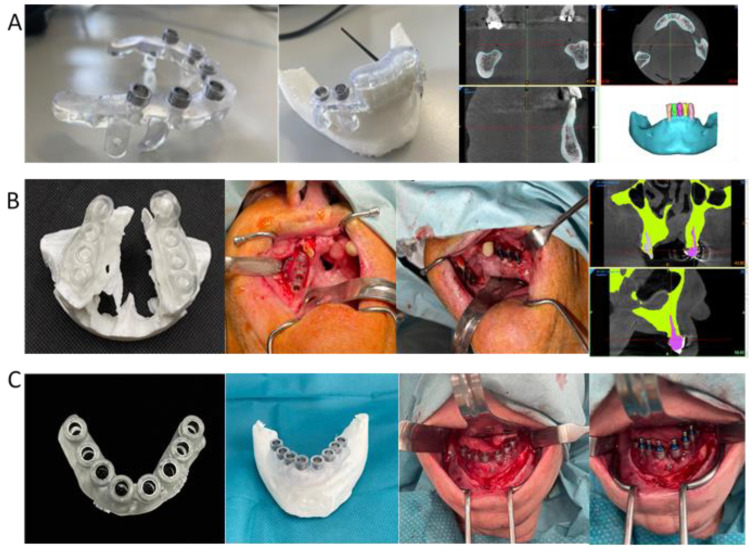
Representative examples of point-of-care (POC) manufacturing of static guided implant surgery templates in three patients (**A**–**C**). Each case illustrates the stages of the digital workflow, including anatomical segmentation and 3D planning, CAD design of the surgical guide, and application on a biomodel or directly in the patient. All guides were designed and produced under the in-house point-of-care manufacturing protocol, ensuring full traceability and quality control in compliance with the European MDR framework.

Average printing duration was 3.8 ± 0.9 h, with a mean resin consumption of 25 ± 5 g per guide. The median design-to-surgery interval was 3 days (range 1–7), and the turnaround time from clinical request to sterilized guide delivery remained consistently under 72 h across the series. No device required external fabrication or reprinting due to non-conformity (Figure 2).

### 3.4. Surgical Variables

The 30 guides were used to place a total of 148 dental implants, corresponding to a mean of 4.9 implants per case (range 2–8). Each implant was positioned according to the prosthetically driven plan and verified intraoperatively through physical fit and postoperative imaging.

Primary stability, assessed by resonance frequency analysis (Osstell^®^), was recorded in 21 guides, yielding a mean ISQ of 79 ± 6—indicating high stability in most cases. Immediate or early loading (≤3 months) occurred in 11 guides (36.7%), mainly in non-irradiated bone with ISQ ≥ 70. Immediate or early loading was restricted to non-irradiated bone. In irradiated segments, a delayed loading protocol (>4 months) was applied regardless of ISQ values. Mean ISQ values showed a similar descriptive range in irradiated (78.5 ± 6.1) and non-irradiated (79.3 ± 5.8) cases.

The mean operative time for implant placement under guided conditions was 54 ± 11 min, representing an average reduction of 20–25% compared with previously recorded freehand cases in the same department. All guides demonstrated stable intraoperative adaptation without fracture or positional misfit.

### 3.5. Postoperative Outcomes

No major intraoperative or postoperative complications were observed. Two guides (6.7%) were associated with minor soft-tissue dehiscence or localized infection, both resolved conservatively.

At a median follow-up of 11 months (range 6–24), 145 implants remained osseointegrated and functional, corresponding to a 98% short-term survival rate. Postoperative CBCT confirmed accurate transfer from the digital plan to the surgical outcome, showing mean coronal deviation of 1.2 ± 0.4 mm, apical deviation of 1.5 ± 0.6 mm, and angular deviation of 3.9° ± 1.2°. Exploratory analysis did not reveal consistent correlations between deviation and guide type, anatomical site, or flap reconstruction. Bone-supported guides in reconstructed jaws showed apical deviation values within expected clinical tolerances (mean 1.6 ± 0.7 mm), overlapping with those observed in tooth-supported guides (1.3 ± 0.4 mm). These values align with reported benchmarks for static guided implant surgery in the literature.

At the descriptive level, short-term outcomes were observed across irradiated and non-irradiated sites, native versus reconstructed bone, and different guide supports, without evident clustering of complications or failures. The few minor complications did not show evident clustering in any specific subgroup. Given the limited size and heterogeneity of these subgroups, no formal statistical comparisons were performed.

**Figure 3:**
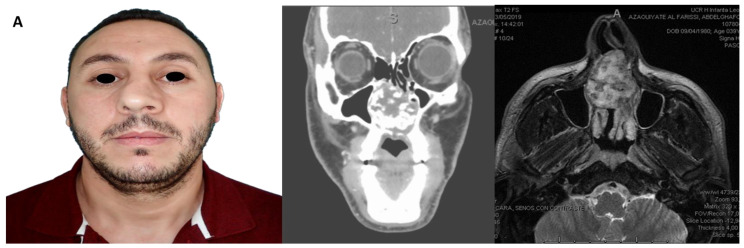

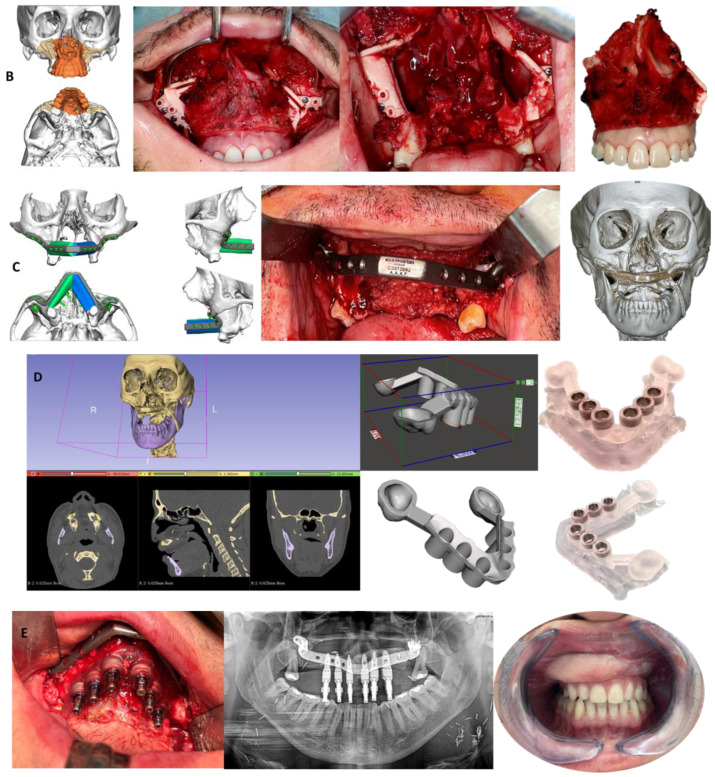
(**A**) Solid mass in the nasal cavity, located in the right paracentral region, diagnosed as a nasal chondrosarcoma. (**B**) 3D planning, virtual design of resection, in-house manufacturing of patient-specific devices and tumor resection. (**C**) Fibular flap harvest using CAD-CAM cutting guides, intraoperative adaptation and fixation of the fibular segments with customized plate and 3D CT postoperative. (**D**) Patient-specific surgical guide fabricated with SLA technology using Biomed Clear V1 material. Surgical guide designed for implant placement through static navigation. (**E**) Placement of six dental implants using static navigation, postoperative control by orthopantomography, and prosthetic rehabilitation within 72 h.

**Figure 4:**
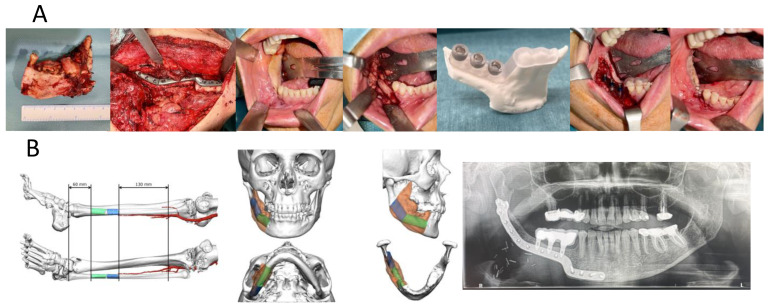
Example of secondary dental implant placement following fibula flap reconstruction. Implant surgery was performed in a second stage after tumor resection and mandibular reconstruction with a patient-specific fibular flap and custom titanium plate. Prosthetic loading was performed under a standard delayed protocol due to moderate ISQ values. (**A**) Surgical images of the tumor specimen, custom mandibular plate reconstruction, and guided implant surgery. (**B**) Radiological images of the virtual surgical planning, and final orthopantomography with the definitive prosthesis.

Functional evaluation showed successful prosthetic rehabilitation in 27 of 30 cases (90%), with restoration of mastication and phonation. Esthetic outcomes were rated ‘good’ or ‘excellent’ in 28 cases (93%), based on clinical records and patient feedback (Figure 3 and Figure 4). A summary of surgical and clinical outcomes is provided in Table 2.

### 3.6. Workflow Efficiency and Learning Curve

Over the four-year period, continuous refinement of the internal workflow—particularly the introduction of predefined design templates and optimized printer parameters—resulted in a 25% reduction in total production time [14]. The iterative collaboration between surgeons and engineers improved both the precision and predictability of implant placement.

No manufacturing nonconformities, sterilization errors, or device-related adverse events were identified. The internal POC traceability system successfully documented every step of the process, ensuring regulatory compliance and reproducibility.

The gradual increase in case volume and complexity reflected an institutional learning curve in which POC-manufactured static guides evolved from isolated applications to an integrated standard for oncologic implant rehabilitation.

## 4. Discussion

This study demonstrates that integrating point-of-care (POC) manufacturing into static guided implant surgery is feasible and effective within a public academic hospital [12,15,20]. In this initial series of 30 guides and 148 implants, static POC-manufactured templates achieved high short-term implant survival (98%), low complication rates, and radiological deviations that were comparable to published computer-assisted implant placement benchmarks. These findings suggest that the POC workflow can reproduce the accuracy and safety expected from computer-guided implant surgery while being integrated into routine oncologic rehabilitation. However, the absence of a control group and the modest sample size warrant cautious interpretation of these outcomes. The steady increase in annual cases from 2021 to 2024 reflects both clinical adoption and organizational consolidation of the UPAM3D workflow [14]. These findings indicate that in-house manufacturing can evolve from a research initiative into a sustainable clinical service for oral cancer rehabilitation.

### 4.1. Comparison with Externalized Manufacturing

Compared with conventional outsourcing to commercial providers, the POC model offers clear logistical and qualitative advantages. The turnaround from clinical request to sterilized delivery was consistently under 72 h, whereas externalized workflows usually require 7–10 days depending on facility capacity and transport logistics. This accelerated process keeps treatment aligned with surgical schedules, which is particularly beneficial in oncologic cases where prosthetic rehabilitation must coincide with adjuvant therapy timelines.

Beyond speed, the in-house workflow promotes direct surgeon–engineer collaboration, allowing iterative modifications to the digital design before fabrication. This teamwork enhances the prosthetically driven approach, which is critical in reconstructed jaws where anatomical landmarks are altered. Previous studies have shown that such immediate feedback improves planning accuracy and surgeon satisfaction, while reducing miscommunication risks common in outsourced settings [1,2]. Managing all stages—from segmentation to sterilization—within the hospital also reinforces traceability and regulatory compliance.

### 4.2. Comparative Advantages of POC Static Navigation Versus Dynamic Navigation Systems in Oncologic Implant Rehabilitation

Static guided surgery and dynamic navigation are complementary strategies for transferring virtual planning to the operative field. Both demonstrate high accuracy—typically 1–2 mm at the implant entry point and <5° of angular deviation—under optimal conditions. However, their clinical, logistical, and economic implications differ considerably, especially in oncologic reconstruction [7,8].

Operationally, POC static navigation offers a rapid, reproducible, and fully traceable workflow. Once the digital plan is approved, in-house design, printing, and sterilization can be completed within 48–72 h. The observed turnaround time of less than 72 h in our series reflects a practical efficiency advantage within our institutional setting, although no formal time or cost comparison with outsourced workflows was performed. By contrast, dynamic navigation systems require intraoperative calibration, optical tracking, and additional setup time—typically adding 20–30 min per procedure. They also depend on line-of-sight tracking, which can be challenging in large maxillofacial defects or multi-surgeon settings.

Economically, the POC static approach is markedly more sustainable in a public academic setting. The mean cost of an in-house printed surgical guide is below 50 €, whereas dynamic systems entail an initial investment exceeding 70,000 € and recurrent maintenance fees. Furthermore, static guides are reusable only in design terms but fully traceable as medical devices under the EU MDR 2017/745 framework, each bearing a documented production batch, resin lot, and sterilization record compliant with ISO 13485 standards [15]. Dynamic navigation, while providing real-time flexibility, does not generate a physical, auditable medical device, limiting traceability in postoperative quality audits.

In terms of accuracy, several comparative studies—including our prior experience in oncologic reconstructions—have shown no statistically significant difference between POC static and dynamic navigation. When postoperative CBCT validation is performed, mean entry deviations around 1.2 mm and apex deviations around 1.5 mm are routinely achieved. The main determinant of precision is therefore not the navigation modality but the rigor of prosthetically driven planning and the mechanical stability of the surgical guide or reference array.

Finally, from an educational and translational perspective, POC manufacturing reinforces the collaboration between surgeons and biomedical engineers, providing hands-on exposure to biomaterials, CAD design, and regulatory processes. This academic independence fosters innovation and scalability while maintaining comparable clinical safety to high-cost dynamic systems. Taken together, these factors justify prioritizing POC static navigation as a cost-effective, auditable, and educationally valuable solution for implant rehabilitation in oral cancer patients (Table 3). Dynamic navigation may still be advantageous in selected complex scenarios—such as cases with limited mouth opening, bulky soft-tissue flaps, or expected intraoperative anatomical changes—where static guides cannot achieve adequate stability. In this context, static and dynamic systems should be viewed as complementary tools within the digital implant armamentarium.

### 4.3. Accuracy and Precision

The geometric accuracy in this series aligns with previous reports on computer-assisted implant placement. Earlier studies reported mean deviations of 1.0–1.5 mm coronally and 1.5–2.0 mm apically for static guides in oncologic reconstructions [3,4,5,7,8]. Our postoperative analyses, when CBCT validation was available, showed similar submillimetric precision (1.2 ± 0.4 mm coronal; 1.5 ± 0.6 mm apical) [7,8]. These accuracy metrics are consistent with previously published data for computer-assisted implant placement, confirming that POC-manufactured static guides can achieve precision levels comparable to industrially fabricated templates.

Such precision is clinically relevant in oral cancer patients, where available bone segments are often limited and implants must be positioned within millimetric proximity to reconstructive plates or vascular pedicles. Together with the high short-term implant survival observed in our cohort, these accuracy values indicate that POC-manufactured static guides can match the performance of industrially manufactured templates in complex oncologic reconstructions, at least in the short term. The observation of consistent accuracy ranges across different guide supports (bone-, tooth-, or mucosa-based) supports the technical robustness of the POC process in this descriptive series. In addition, the ability to fabricate patient-specific guides under a validated ISO 13485 framework ensures quality assurance comparable to that of industrial manufacturing.

The results confirm that POC manufacturing can achieve the same precision standards as externalized workflows, provided that digital planning, printer calibration, and sterilization are tightly controlled. This finding is consistent with previous comparative analyses demonstrating no statistically significant difference in accuracy between industrially manufactured and in-house printed guides when identical design and validation protocols are followed [6,7,8].

### 4.4. Integration of ISQ and Early Loading

Objective assessment of implant stability is crucial for prosthetic planning, particularly in irradiated or reconstructed bone. In this series, resonance frequency analysis (RFA) quantified implant stability using the Implant Stability Quotient (ISQ), with a mean value of 79 ± 6 [22,23,24,25]. This parameter guided immediate or early loading (≤3 months) in over one third of the cases [26,27].

Comparable experiences, such as the “Jaw-in-a-Day” protocol [6], reported immediate loading with ISQ ≥ 84 without postoperative complications. Incorporating ISQ measurement into routine workflows offers several advantages: it standardizes the definition of “primary stability,” facilitates interinstitutional comparisons, and supports objective decision-making. Furthermore, documenting ISQ values in POC registries allows continuous quality monitoring and predictive modeling of implant success in oncologic populations.

The standardization of this metric should therefore be considered a priority in future multicenter collaborations, where ISQ could serve as a common endpoint to evaluate the influence of flap type, radiation, and loading protocol on long-term outcomes [28].

### 4.5. Sustainability and Educational Impact

Beyond clinical performance, the POC manufacturing model enhances institutional sustainability and self-sufficiency. By reducing reliance on commercial suppliers, hospitals can optimize resources and maintain continuity of care during supply chain disruptions. In our institutional setting, the per-device material cost was approximately €50 per guide, which was lower than reported prices for outsourced production. However, no formal cost-effectiveness analysis was performed, and these economic observations should be regarded as institution-specific and preliminary, rather than generalizable.

In addition, the POC framework inherently supports training and translational research. The collaboration between surgeons, prosthodontists, and biomedical engineers within the same institution promotes interdisciplinary education and accelerates innovation cycles. Resident involvement in segmentation, design validation, and quality control offers practical exposure to digital manufacturing processes that are increasingly essential in contemporary surgical practice [9].

From a regulatory perspective, POC manufacturing complies with the provisions of the European Medical Device Regulation (MDR) 2017/745, which specifically recognizes the right of healthcare institutions to manufacture custom devices for their own patients under defined quality standards. The UPAM3D workflow fulfills these requirements through a certified ISO 13485 quality management system, ensuring full traceability of each device from digital design to sterilization.

### 4.6. Limitations and Future Directions

This study has several limitations. Its retrospective design and modest sample size preclude statistical significance testing and multivariate analysis of outcome predictors. Moreover, detailed ISQ measurements and quantitative deviation data were unavailable for some cases, reflecting the variability inherent to real-world clinical practice. The retrospective, single-center, consecutive-case design was chosen as an initial real-world evaluation of the newly implemented POC workflow, and the results should therefore be interpreted as exploratory and hypothesis-generating rather than as definitive evidence of superiority over alternative techniques.

Nevertheless, the descriptive evidence supports the clinical reliability of POC-manufactured guides in oncologic implant rehabilitation. Future prospective and multicenter studies are warranted to validate these findings under standardized reporting protocols. Integration of automated accuracy evaluation tools, digital twins, and AI-based error prediction models may further enhance reproducibility and workflow optimization. The UPAM3D unit is currently participating in the development of a multicenter collaborative registry coordinated by the National Network for Advanced Manufacturing in Healthcare. This initiative will collect standardized accuracy, cost, and patient-outcome data from multiple university hospitals, providing a larger evidence base for validation of the POC-guided implant model.

Furthermore, no formal cost-effectiveness analysis was conducted. The economic and organizational benefits discussed in this study are based on internal material cost estimates and published data on commercial manufacturing timelines, and should be interpreted as indicative rather than conclusive. Accordingly, any statements regarding workflow efficiency or economic aspects should be interpreted in the context of our single-center institutional experience.

Another avenue for development is the harmonization of material testing and sterilization validation across European academic manufacturing units. Establishing shared repositories of digital designs, resins, and quality control templates would facilitate benchmarking and cross-institutional audits, ultimately strengthening patient safety and regulatory transparency.

### 4.7. Broader Implications

These findings demonstrate that high-quality, patient-specific surgical devices can be safely and efficiently produced within a public hospital. This paradigm democratizes access to precision implant rehabilitation, which was previously restricted to centers relying on commercial outsourcing or proprietary navigation systems.

By embedding POC manufacturing into the surgical workflow, institutions not only expand clinical capabilities but also build local resilience and innovation capacity. In this sense, POC represents not merely a technological advancement but a structural evolution toward a more autonomous, agile, and patient-centered healthcare model.

## 5. Conclusions

Static guided implant surgery supported by point-of-care (POC) manufacturing can be safely and effectively implemented within a public university hospital. The UPAM3D workflow allows the design, fabrication, and sterilization of patient-specific surgical guides entirely in-house, providing a fast, reproducible, and auditable pathway for implant rehabilitation in oral cancer patients.

The clinical results—148 implants placed using 30 POC-manufactured guides—show accuracy, high short-term implant survival, and a low rate of minor complications. The standardized use of biocompatible resins, ISO 13485-validated sterilization, and digital traceability ensure regulatory compliance equivalent to that of industrially produced devices.

From an operational perspective, POC manufacturing shortens turnaround time from several days to less than 72 h and enhances interdisciplinary collaboration between surgeons and biomedical engineers. These efficiencies make the model sustainable and scalable for other high-complexity surgical specialties.

Functionally, the integration of objective metrics such as the Implant Stability Quotient (ISQ) supports consistent decision-making for immediate or early loading, particularly in reconstructed or irradiated bone.

Future directions should focus on multicenter validation of POC protocols, harmonization of material testing standards, and the creation of shared registries to monitor long-term outcomes. Overall, the adoption of hospital-based POC manufacturing represents a tangible step toward a more precise, efficient, and self-sufficient model of oncologic implant rehabilitation. This work should therefore be interpreted as a feasibility and implementation study of a point-of-care guided implant workflow, and further comparative studies with larger cohorts and longer follow-up are required to assess long-term outcomes and relative effectiveness.

## Figures and Tables

**Table 1:** Baseline characteristics and POC workflow parameters (n = 30).

Variable	Description/Category	n (%)/Median (Range)	Notes
Year of surgery	2021/2022/2023/2024	2/6/6/16	Progressive adoption of POC workflow
Anatomical site	Maxilla/Mandible	21/14	Some involved both maxilla and mandible
Diagnosis	SCC/Ameloblastoma/Other benign	18/9/3	Oral cavity oncologic reconstructions
Reconstruction type	Fibula/Iliac/Native	20/2/8	Based on surgical notes
Radiotherapy	Yes/No	9 (30%)/21 (70%)	≥50 Gy in irradiated patients
Type of guide	Bone-/Tooth-/Mucosa-supported	18/7/5	Designed prosthetically driven
Resin used	BioMed Clear/Dental SG/LT Clear	17/10/3	Formlabs Form 3B/3BL SLA printer
Printing technology	SLA (stereolithography)	—	100% in-house (UPAM3D)
Sterilization method	Steam (134 °C, 5 min)	—	ISO 13485 validated process
Externalization	None	0 (0%)	All printed in-house
Engineer–surgeon feedback loops	≥2 per case	30 (100%)	Documented in UPAM3D registry
Planning software	Blender/IPS CaseDesigner/3-matic	—	Used depending on case complexity
Design-to-surgery time	Median 3 days (1–7)	—	Request → sterile guide ready

**Table 2:** Surgical and clinical outcomes of static guided implant surgery (n = 30).

Variable	n (%)/Mean ± SD/Median (Range)	Comment
No. of implants per case	3 (1–6)	Derived from operative notes
Primary stability (ISQ)	79 ± 6 (when recorded)	High stability in most cases
Immediate/early loading	11 (36.7%)	Based on ISQ ≥ 70 and prosthetic readiness
Radiotherapy exposure	9 (30%)	Mostly in mandibular reconstructions
Reconstruction flap	20 (66%) fibula/2 (6.6%) iliac	From surgical reports
Intraoperative deviation	1.2 ± 0.4 mm (coronal); 1.5 ± 0.6 mm (apical)	Comparable to literature values
Surgical complications	2 (6.7%) minor (dehiscence/infection)	Managed conservatively
Implant survival (<6 mo)	148/148 (100%)	No early losses
Time-to-prosthesis	95 days (45–210)	Longer in irradiated patients
Follow-up duration	11 months (6–24)	Ongoing registry follow-up
Functional outcome	27 (90%) normal mastication	Post-prosthetic evaluation
Esthetic satisfaction (self-reported)	28 (93%) “good–excellent”	From clinical notes

Notes: Units are expressed consistently; ISQ is unitless; deviations in millimeters.

**Table 3:** Comparative aspects between POC Static Guided Surgery and Dynamic Navigation.

Aspect	POC Static Guided Surgery	Dynamic Navigation
Workflow	Rapid, reproducible, minimal intraoperative setup	Flexible, real-time adaptable
Precision	1–2 mm deviation (validated)	1–2 mm deviation (dependent on tracking)
Cost-efficiency	Low (≤50 € per guide)	High initial and maintenance costs
Regulatory traceability	Full ISO 13485 traceability	Limited (non-device-based)
Suitability for oncology	Excellent for complex resections and flap-based reconstructions	Ideal for secondary implant placement with variable anatomy

## Data Availability

The data presented in this study are available on request from the corresponding author.

## Abbreviations

The following abbreviations are used in this manuscript:

POC	Point-of-Care
UPAM3D	Advanced Planning and 3D Manufacturing Unit
ISO 13485	Medical devices quality management system
ISO 17665	Sterilization of health care products by moist heat
EU MDR 2017/745	European Union Medical Device Regulation
CE	Conformité Européenne
CT	Computed Tomography
CBCT	Cone-Beam Computed Tomography
DICOM	Digital Imaging and Communications in Medicine
VSP	Virtual Surgical Planning
CAD/CAM	Computer-Aided Design/Computer-Aided Manufacturing
AM	Additive Manufacturing
SLA	Stereolithography
UV	Ultraviolet
ISQ	Implant Stability Quotient
RFA	Resonance Frequency Analysis
STROBE	Strengthening the Reporting of Observational Studies in Epidemiology
SPSS	Statistical Package for the Social Sciences
SD	Standard Deviation
SCC	Squamous Cell Carcinoma
Gy	Gray
